# Frailty and pre-frailty in middle-aged and older adults and its association with multimorbidity and mortality: a prospective analysis of 493 737 UK Biobank participants

**DOI:** 10.1016/S2468-2667(18)30091-4

**Published:** 2018-06-14

**Authors:** Peter Hanlon, Barbara I Nicholl, Bhautesh Dinesh Jani, Duncan Lee, Ross McQueenie, Frances S Mair

**Affiliations:** a General Practice and Primary Care, Institute of Health and Wellbeing, University of Glasgow, 1 Horselethill Road, Glasgow, G12 9LX, Scotland, United Kingdom; b School of Mathematics and Statistics, University of Glasgow, Glasgow, UK

## Abstract

**Background:**

Frailty is associated with older age and multimorbidity (two or more long-term conditions); however, little is known about its prevalence or effects on mortality in younger populations. This paper aims to examine the association between frailty, multimorbidity, specific long-term conditions, and mortality in a middle-aged and older aged population.

**Methods:**

Data were sourced from the UK Biobank. Frailty phenotype was based on five criteria (weight loss, exhaustion, grip strength, low physical activity, slow walking pace). Participants were deemed frail if they met at least three criteria, pre-frail if they fulfilled one or two criteria, and not frail if no criteria were met. Sociodemographic characteristics and long-term conditions were examined. The outcome was all-cause mortality, which was measured at a median of 7 years follow-up. Multinomial logistic regression compared sociodemographic characteristics and long-term conditions of frail or pre-frail participants with non-frail participants. Cox proportional hazards models examined associations between frailty or pre-frailty and mortality. Results were stratified by age group (37–45, 45–55, 55–65, 65–73 years) and sex, and were adjusted for multimorbidity count, socioeconomic status, body-mass index, smoking status, and alcohol use.

**Findings:**

493 737 participants aged 37–73 years were included in the study, of whom 16 538 (3%) were considered frail, 185 360 (38%) pre-frail, and 291 839 (59%) not frail. Frailty was significantly associated with multimorbidity (prevalence 18% [4435/25 338] in those with four or more long-term conditions; odds ratio [OR] 27·1, 95% CI 25·3–29·1) socioeconomic deprivation, smoking, obesity, and infrequent alcohol consumption. The top five long-term conditions associated with frailty were multiple sclerosis (OR 15·3; 99·75% CI 12·8–18·2); chronic fatigue syndrome (12·9; 11·1–15·0); chronic obstructive pulmonary disease (5·6; 5·2–6·1); connective tissue disease (5·4; 5·0–5·8); and diabetes (5·0; 4·7–5·2). Pre-frailty and frailty were significantly associated with mortality for all age strata in men and women (except in women aged 37–45 years) after adjustment for confounders.

**Interpretation:**

Efforts to identify, manage, and prevent frailty should include middle-aged individuals with multimorbidity, in whom frailty is significantly associated with mortality, even after adjustment for number of long-term conditions, sociodemographics, and lifestyle. Research, clinical guidelines, and health-care services must shift focus from single conditions to the requirements of increasingly complex patient populations.

**Funding:**

CSO Catalyst Grant and National Health Service Research for Scotland Career Research Fellowship.

## Introduction

Multimorbidity, the presence of two or more long-term conditions, is an increasing clinical and public health challenge.^1^ Multimorbidity has been shown to be linked to, although not synonymous with, frailty.^2^ Evidence informing management of multimorbidity remains sparse with most guidelines focusing on single diseases. Both the National Institute for Health and Care Excellence (NICE) and British Geriatrics Society emphasise the importance of recognising frailty to identify patients with multimorbidity who are at greater risk of adverse outcomes and who might benefit from treatment optimisation.^3^, ^4^ The term frailty describes impaired resolution to homoeostasis following a stressor event.^5^ Frailty has been found to be a predictor of mortality, falls, worsening disability, hospitalisation, and care home admission in cohorts of elderly people.^5^ Various frailty classifications exist, but most widely cited is the frailty phenotype described by Fried and colleagues,^6^ which defines frailty as the presence of three or more out of five indicators: weakness (reduced grip strength), slowness (gait speed), weight loss, low physical activity, and exhaustion. People with one or two indicators are classified as pre-frail. This classification has been adapted for various epidemiological cohorts.^7^, ^8^ Fried and colleagues conceptualised frailty as a distinct clinical entity causing disability independently of clinical and subclinical disease, but frailty is widely seen as both a predictor and outcome of multimorbidity.^9^

Although the prevalence of multimorbidity and frailty increases with age, neither is limited to the elderly. Although most people over the age of 65 years have multimorbidity, the absolute number of people with multiple conditions is greater in people younger than age 65 years.^1^ Although the original description of the frailty phenotype excluded participants younger than age 65 years,^6^ frailty can also affect younger people.^2^, ^10^, ^11^ Frailty reflects biological and phenotypic, rather than chronological, age.^10^, ^11^ Precursors of frailty tend to arise earlier in the life-course.^10^ Implicit in the frailty phenotype is progression from pre-frailty to frailty;^6^ however, this progression could be reversible in some patients.^12^ Frailty predicts future disability but might be modifiable, particularly at an early stage,^12^ suggesting that identification of frailty and pre-frailty in middle age (those younger than 65 years) might have implications for prognosis, optimising care, and planning interventions, particularly in individuals with multimorbidity.

**Research in context**

**Evidence before this study**

We searched Ovid MEDLINE between January, 1996, and December, 2017, without language restriction, for articles examining the association between frailty or multimorbidity and mortality in people aged younger than 65 years. We used the following terms: “frail*”, “frail elderly”, “middle-age*”, “multimorbid*”, “multi-morbid*”, “multimorbidity”, “mortality”, “death”, and “survival”. Searches were supplemented by hand-searching reference lists of any identified systematic reviews. We also searched for articles citing the original description of the “frailty phenotype” by Fried and colleagues. We included any community-based studies with longitudinal follow-up assessing mortality that included any participants aged younger than 65 years. Thirteen cohorts were identified that included some measure of frailty and included those aged younger than 65 years (three of these used the frailty phenotype model). In all but one of these studies, the number of participants aged younger than 65 years was unclear and the mean age of the frail participants was more than 70 years. Only one study (n=998) specifically assessed the impact of frailty on mortality in those aged younger than 65 years. The phenotype model did not show a statistically significant association with mortality. No studies of the effects of multimorbidity on mortality included an assessment of frailty.

**Added value of this study**

Our findings show a greater than two times increase in 7-year mortality that is associated with frailty (assessed using the frailty phenotype model) in a large cohort of 493 737 people aged 37–73 years. These findings are consistent across age and sex strata (except in women aged 37–45 years), and are independent of the mortality risk associated with multimorbidity. To our knowledge, this is the largest study to assess the effect of frailty on mortality to date. It also includes a greater proportion of participants aged younger than 65 years than previous studies of frailty. Previous studies have not assessed the association between the frailty phenotype and mortality adjusting for associated multimorbidity. Previous studies of frailty have also not adjusted for sociodemographics or lifestyle. Our study shows that frailty was strongly associated with socioeconomic deprivation, obesity, smoking, and multimorbidity and that the effect on mortality persisted after adjusting for these factors.

**Implications of all the available evidence**

The management of frailty is an important clinical priority for all health professionals, not just those caring for older people. Frailty is identifiable in middle-aged as well as older adults, and is associated with mortality independently of the extent of multimorbidity as well as age, sex, sociodemographic, and lifestyle factors. Frailty is also associated with a range of long-term conditions. Clinical guidelines must emphasise the importance of identifying and addressing frailty in a range of clinical contexts. Future research needs to focus on the clinical implications of frailty across different disease contexts and broader age ranges, to inform the accurate and efficient identification of frailty in clinical settings, and to inform development of interventions to modify frailty and ameliorate its effects earlier in the life-course. Our findings suggest an urgent need to re-imagine and reconfigure health-care services to better meet the needs of our increasingly complex patient populations.

Despite the potential importance of frailty in middle-aged people, most epidemiological studies assessing frailty have excluded those aged younger than 65 years.^4^, ^8^ Methods to identify frailty in clinical practice, as well as interventions targeting people with frailty, have almost exclusively concerned people aged older than 65 years.^4^, ^5^ The few epidemiological studies of frailty that include people younger than 65 years (with lower age limits ranging from 50–65 years) generally have small sample sizes, with most participants being aged over 65 years.^13^, ^14^, ^15^, ^16^, ^17^, ^18^, ^19^ Analyses of the effect on mortality at different ages are limited to a few studies, and none have adjusted for lifestyle factors, socioeconomic deprivation, or multimorbidity. The prognostic significance of frailty in people younger than 65 years, and its association with specific patterns of multimorbidity, remains uncertain. This study aims to assess if an adaptation of the frailty phenotype using data from UK Biobank (a large community cohort of over half a million people aged 37–73 years) can be used to identify people at increased risk of mortality across a range of ages; to quantify the association between frailty, multimorbidity, and long-term conditions; and to assess the effect of frailty and pre-frailty on all-cause mortality, adjusting for multi-morbidity, sociodemographic characteristics, and lifestyle factors.

## Methods

### Study design and participants

This was a prospective, population-based cohort study of participants enrolled in the UK Biobank. A total of 502 640 participants were recruited by postal invitation between 2006 and 2010 (5% response rate) and attended one of 22 assessment centres across England, Scotland, and Wales. Each participant completed a touchscreen questionnaire, a nurse-led interview, and had physical measurements. All participants gave written informed consent for data collection, analysis, and linkage. Participants had to be registered with a general practitioner, live within reasonable travelling distance of the assessment centre, and aged 40–69 years to be invited to participate in the study. In practice, some outside the age limit were also included. This study had ethical approval and is part of UK Biobank project 14151 (NHS National Research Ethics Service 16/NW/0274).

### Procedures

Participants reported their long-term conditions at baseline assessment. The list of morbidities described in this paper was taken from a list of 43 long-term conditions originally established for a large epidemiological study in Scotland, through systematic review, the Quality and Outcomes Framework, NHS Scotland, and an expert panel,^1^ and subsequently amended for UK Biobank.^20^ The number of long-term conditions reported was summed and multimorbidity categorised as zero, one, two, three, or at least four long-term conditions.

Age was categorised as 37–45, 45–55, 55–65, and 65–73 years. Townsend scores were derived from participant postcodes as an area-specific measure of socioeconomic deprivation based on preceding national census data (percentage unemployment, percentage car ownership, percentage home ownership, and household overcrowding).^21^, ^22^ Body-mass index was categorised into underweight (<18·5 kg/m^2^), normal weight (≥18·5–25 kg/m^2^), overweight (>25–30 kg/m^2^), and obese (>30 kg/m^2^). Smoking was categorised as current smoker, ex-smoker, and non-smoker. Alcohol intake was based on self-reported frequency of alcohol intake (never or special occasions only; one to three times per month; one to four times per week; or daily or almost daily). Grip strength was assessed using a Jamar J00105 hydraulic hand dynamometer. Right and left hand measurements were recorded, and the higher of the two used in this analysis. Other variables included in the frailty phenotype were slow walking speed, exhaustion, recent weight loss, and physical activity, which were defined based on assessment centre questionnaire (table 1).

**Table 1:** Frailty criteria

	**Cardiovascular Health Study**^6^	**UK Biobank**
Weight loss	Self-reported: “In the last year, have you lost more than 10 pounds unintentionally?” (response: yes=1, no=0)	Self-reported: “Compared with one year ago, has your weight changed?” (response: yes, lost weight=1, other=0)*
Exhaustion	Self-reported (CES depression scale, two questions): “How often in the last week (a) did you feel that everything was an effort, or (b) could you not get going?” (response: moderate amount of the time [3–4 days] or most of the time=1, other=0)	Self-reported: “Over the past two weeks, how often have you felt tired or had little energy?” (response: more than half the days or nearly every day=1, other=0)*
Physical activity	Self-reported: Minnesota Leisure Time Activity Questionnaire (18 items). Kcal of activity per week estimated, and the lowest 20% were identified as meeting frail criteria	Self-reported: UK Biobank physical activity questionnaire. We classified the responses into: none (no physical activity in the last 4 weeks), low (light DIY activity [eg, pruning, watering the lawn] only in the past 4 weeks), medium (heavy DIY activity [eg, weeding, lawn mowing, carpentry and digging], walking for pleasure, or other exercises in the past 4 weeks), and high (strenuous sports in the past 4 weeks) (response: none or light activity with a frequency of once per week or less=1, medium or heavy activity, or light activity more than once per week=0)†
Walking speed	Measured time to walk 15 feet	Self-reported: “How would you describe your usual walking pace?” (response: slow=1, other=0)*
Grip strength	Measured grip strength, adjusted for sex and body-mass index (lowest 20% of cohort identified as meeting frail criteria)	Measured grip strength (sex and body-mass index adjusted cutoffs taken from Fried and colleagues^6^)‡

Criteria were adapted from Fried and colleagues^6^ and a comparison is shown with those used in the Biobank study.

* Approximation based on available variables in UK Biobank assessment centre data.

† Definition used in SHARE adaptation of the frailty phenotype.^19^

‡ Definition used in original description by Fried and colleagues.^6^

Frailty status was assessed using the five frailty phenotype indicators originally described by Fried and colleagues.^6^ As specific questions and measurements gathered for UK Biobank differed from the Cardiovascular Health Study on which the phenotype is based, we adapted the definitions of the criteria to be estimable using available data. Table 1 shows our definitions alongside those of Fried and colleagues. When possible, these adaptations were based on previously validated versions of the phenotype.^7^, ^8^, ^19^

Participants were classified as not frail (met none of the frailty criteria), pre-frail (met one or two criteria), or frail (met three or more criteria) according to cutoffs described by Fried and colleagues, to ensure consistency with the existing literature.^6^ Participants with missing data for one or more frailty criteria were excluded.

### Outcomes

The primary outcome was all-cause mortality. Data from the UK Biobank baseline assessment centre were linked to national mortality records by UK Biobank. Median follow-up duration was 7 years (IQR 76–93 months).

### Statistical analysis

All analyses were planned before inspection of the data in keeping with STROBE guidelines.

First, sociodemographic characteristics were sum-marised for frail, pre-frail, and non-frail participants. Age, sex, ethnic origin, socioeconomic status (Townsend score), smoking status, body-mass index, frequency of alcohol intake, number of long-term conditions, and number of medications were compared using χ^2^ test for categorical variables, and Kruskal-Wallis test for continuous variables.

Next, we constructed adjusted models to test the association between sociodemographic characteristics and frailty status. We used a multinomial logistic regression model that allowed simultaneous estimation of the probability of different outcomes. Separate odds ratios (OR) and 95% CI were calculated for the frail and pre-frail groups, comparing each with the reference group (non-frail). All significant variables from the descriptive analysis were included in the final model except ethnic origin (because of an insufficient number of non-white participants for meaningful results).

Next, we assessed the association between long-term conditions and frailty status. Multinomial logistic regression models were fitted (using non-frail as the reference group) to assess the odds of frailty and pre-frailty in the context of each long-term condition. Separate models were fitted for each long-term condition with a prevalence of greater than 1% in the cohort, adjusting for age, sex, deprivation, body-mass index, smoking status, and alcohol use. To compensate for multiple testing we calculated 99·75% CI based on a Bonferroni correction.

To assess the validity of the criteria used in our frailty definition we assessed the effect of each individual variable on all-cause mortality using separate Cox proportional hazards models. Men and women, as well as age categories (37–45, 45–55, 55–65, and 65–73 years), were modelled separately to assess if the mortality risk differed across age ranges and between sexes. All variables were then included in a combined model, adjusted for age and sex, to assess the independent effect of each variable. The incremental effect of increasing number of frailty indicators was assessed by comparing the hazard ratios for the presence of one, two, three, four, or five indicators, using the non-frail as the reference group. This was done because the cutoffs of meeting the pre-frail and frail criteria were validated on an older population.

Finally, Cox-proportional hazards models were fitted to assess the effect of frailty status (non-frail, pre-frail, or frail) on all-cause mortality. Models were stratified by age and sex, and adjusted for socioeconomic status, body-mass index, smoking status, alcohol frequency, and multimorbidity count. Results were presented as hazard ratios (HR) and 95% CI for each age and sex stratum. The following subgroup analyses (also stratified by age and sex) were done along with tests for statistical interactions between predictors and frailty, to assess if the effect of frailty was modified by other covariates: one or fewer long-term conditions versus at least two long-term conditions, never-smokers versus current or previous smokers, never or occasional alcohol drinkers versus those drinking weekly or more frequently, body-mass index of more than 25 versus body-mass index of 25 or lower, and more affluent socioeconomic status versus more deprived status (divided at median Townsend score).

All analyses were done using R software (version 3.4.1). Descriptive or unadjusted analyses included all available data for eligible participants. Adjusted analyses excluded those with missing data for one or more covariates. Syntax for the generation of derived variables and for the analysis used for this study will be submitted to UK Biobank for record.

### Role of the funding source

The funder of the study had no role in study design, data collection, data analysis, data interpretation, or writing of the report. All authors had full access to all the data in the study and the corresponding author had final responsibility for the decision to submit for publication.

## Results

Of the 502 640 participants recruited to UK Biobank, 493 737 (98%) had complete data for all five frailty indicators and were included in the current study; of these, 266 618 (54%) were female, 399 481 (81%) were aged younger than 65 years, 16 538 (3%) met the criteria for frailty and 185 360 (38%) were pre-frail. Prevalence of frailty status for each age and sex category is shown in table 2. The prevalence of frailty and pre-frailty increased with age. Low grip strength and slow walking pace were associated with increasing age, whereas self-reported exhaustion and weight loss were more prevalent in younger participants.

**Table 2:** Frailty criteria and frailty status

	**Total (n=493 737)**	**Women**				**Men**			
		37–45 years (n=34 218)	45–55 years (n=81 144)	55–65 years (n=116 759)	65–73 years (n=36 623)	37–45 years (n=28 993)	45–55 years (n=62 906)	55–65 years (n=97 239)	65–73 years (n=35 855)
**Frailty indicators**									
Low grip strength	64 190 (13%)	1898 (6%)	7625 (9%)	19 070 (16%)	8432 (23%)	1817 (6%)	5196 (8%)	12 811 (13%)	7341 (21%)
Weight loss	74 603 (15%)	5870 (17%)	12 666 (16%)	17 880 (15%)	5287 (14%)	4697 (16%)	7411 (15%)	14 021 (14%)	4771 (13%)
Slow walking pace	39 903 (8%)	1666 (5%)	5559 (7%)	10 496 (9%)	4383 (12%)	1187 (4%)	3562 (6%)	8816 (9%)	4234 (12%)
Exhaustion	60 407 (12%)	6131 (18%)	13 787 (17%)	13 421 (12%)	3582 (10%)	3808 (13%)	7750 (12%)	9166 (9%)	2762 (8%)
Low physical activity	42 696 (9%)	3125 (9%)	7917 (10%)	10 468 (9%)	3662 (10%)	2294 (8%)	5282 (8%)	7582 (8%)	2366 (7%)
**Frailty status**									
Non-frail (zero frailty indicators)	291 839 (59%)	20 311 (59%)	47 222 (58%)	66 643 (57%)	19 393 (53%)	18 222 (63%)	39 602 (63%)	59 856 (62%)	20 590 (57%)
Pre-frail (one or two frailty indicators)	185 360 (38%)	12 988 (38%)	31 109 (38%)	45 660 (39%)	15 467 (42%)	10 273 (35%)	21 747 (35%)	34 151 (35%)	13 965 (39%)
Frail (three or more frailty indicators)	16 538 (3%)	919 (3%)	2813 (4%)	4456 (4%)	1763 (5%)	498 (2%)	1557 (3%)	3232 (3%)	1300 (4%)

χ^2^ test for trend: p<0·0001 for all variables.

Table 3 shows adjusted multinomial logistic regression models comparing baseline characteristics for frail and pre-frail participants with a reference group of non-frail participants, excluding 7191 (1%) of 493 737 participants who had missing data for one or more covariates. The unadjusted baseline characteristics for participants with each frailty status are shown in the appendix. Participants with frailty were more likely to be female. A higher proportion of frail participants were relatively socio-economically deprived (6952 [42%] of 16 538 frail participants in the most deprived quintile *vs* 45 971 [16%] of 291 839 in the non-frail group; OR 3·71, 95% CI 3·49–3·94). Frail participants were more likely to smoke (3312 frail participants [20%] were current smokers *vs* 25 888 non-frail participants [9%]; 2·47, 2·36–2·60), to be non-drinkers or occasional drinkers (7610 frail participants [46%] were never-drinkers *vs* 42 766 non-frail participants [15%]; 3·09, 2·97–3·22), and were more likely to be obese (8279 frail participants [52%] had a body-mass index >30 *vs* 53 218 non-frail participants [18%]; 4·10, 3·90–4·31) or be underweight (150 frail participants [1%] *vs* 1371 non-frail participants [<1%]; 2·92, 2·41–3·53). Furthermore, when considering the pre-frail group, the percentages of each of these characteristics (deprivation, smoking, obesity, and non-drinker) were higher than in the non-frail group, but lower than those observed in the frail group (appendix).

**Table 3:** Multivariate adjusted association between sociodemographic characteristics and frailty status

	**Pre-frail *vs* non-frail**	**Frail *vs* non-frail**
**Sex**		
Male	1 (ref)	1 (ref)
Female	1·19 (1·17–1·20)	1·21 (1·17–1·26)
**Age**		
37–<45 years	1 (ref)	1 (ref)
45–<55 years	0·96 (0·94–0·98)	1·15 (1·08–1·23)
55–<65 years	0·93 (0·91–0·94)	1·13 (1·06–1·20)
65–73 years	1·00 (0·98–1·03)	1·18 (1·10–1·27)
Socioeconomic deprivation quintile*		
1	1 (ref)	1 (ref)
2	1·05 (1·03–1·07)	1·19 (1·10–1·27)
3	1·15 (1·13–1·17)	1·48 (1·38–1·58)
4	1·28 (1·25–1·30)	2·07 (1·94–2·21)
5	1·62 (1·59–1·65)	3·71 (3·49–3·94)
**Smoking status**		
Never	1 (ref)	1 (ref)
Previous	1·05 (1·03–1·06)	1·07 (1·03–1·12)
Current	1·42 (1·39–1·45)	2·47 (2·36–2·60)
**Alcohol frequency**		
Daily	0·88 (0·87–0·90)	0·75 (0·71–0·80)
1–4 times per week	1 (ref)	1 (ref)
1–3 times per month	1·23 (1·20–1·25)	1·48 (1·40–1·57)
Occasional or never	1·59 (1·56–1·61)	3·09 (2·97–3·22)
**Body-mass index**		
≤18·5	1·48 (1·36–1·61)	2·92 (2·41–3·53)
>18·5–25	1 (ref)	1 (ref)
>25·0–30	1·35 (1·33–1·37)	1·51 (1·42–1·59)
>30	2·18 (2·14–2·22)	4·10 (3·90–4·31)
**Multimorbidity**		
No long-term conditions	1 (ref)	1 (ref)
One long-term condition	1·32 (1·30–1·34)	2·27 (2·12–2·42)
Two long-term conditions	1·72 (1·69–1·75)	5·12 (4·80–5·47)
Three long-term conditions	2·25 (2·20–2·31)	10·4 (9·69–11·1)
At least four long-term conditions	3·31 (3·21–3·42)	27·1 (25·3–29·1)

The model used was adjusted for age (categorical), sex, socioeconomic status (Townsend score), smoking status, alcohol use frequency, body-mass index, and multimorbidity count. The results are based on n=488 087 participants with complete data for all covariates (5650 [1%] of 493 737 had missing data and were excluded).

* Socioeconomic deprivation quintile scale indicates those that are the least (1) and most (5) deprived.

**Figure 1:**
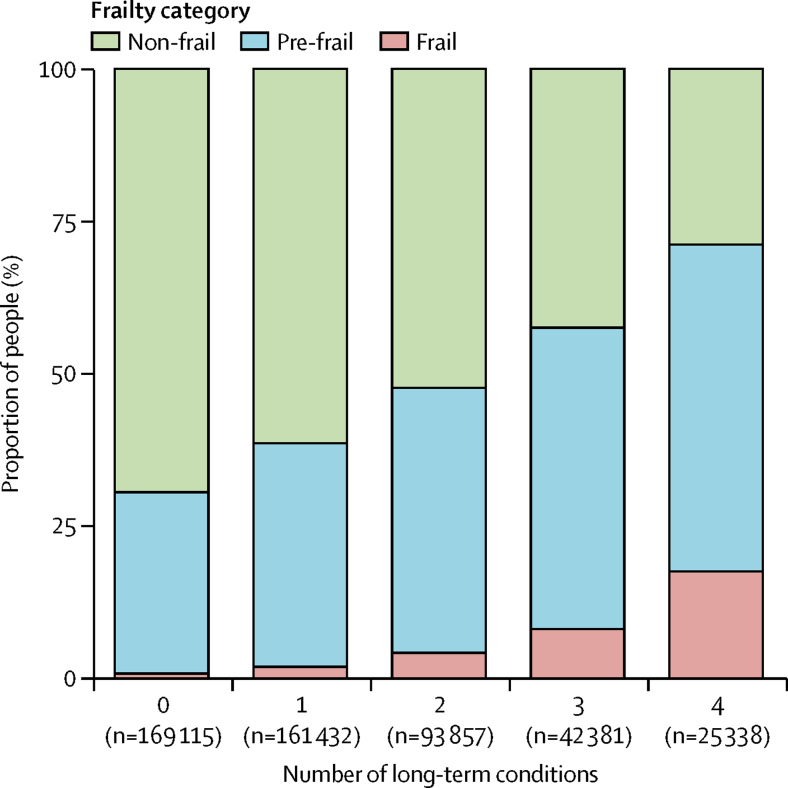
Prevalence of frailty and pre-frailty categorised by number of long-term conditions

The prevalence of frailty at different levels of multimorbidity is shown in figure 1. The prevalence of both frailty and pre-frailty increased with increasing multimorbidity. Of the 161 576 participants with at least two long-term conditions, 11 865 (7%) met the criteria for frailty. Multimorbidity was also more common in frail participants: 11 865 (72%) of 16 538 frail participants were multimorbid compared with 66 022 (25%) of 291 839 non-frail participants (OR 5·14, 95% CI 4·82–5·49 for two long-term conditions; 10·4, 9·7–11·2 for three long-term conditions). For those with at least four long-term conditions, frailty was prevalent in 4435 (18%) of 25 338 participants. The proportion with at least four long-term conditions was 4435 (27%) of 16 538 in the frail group versus 7299 (2·5%) of 291 839 in the non-frail group (OR 27·1, 95% CI 25·3–29·1).

**Figure 2:**
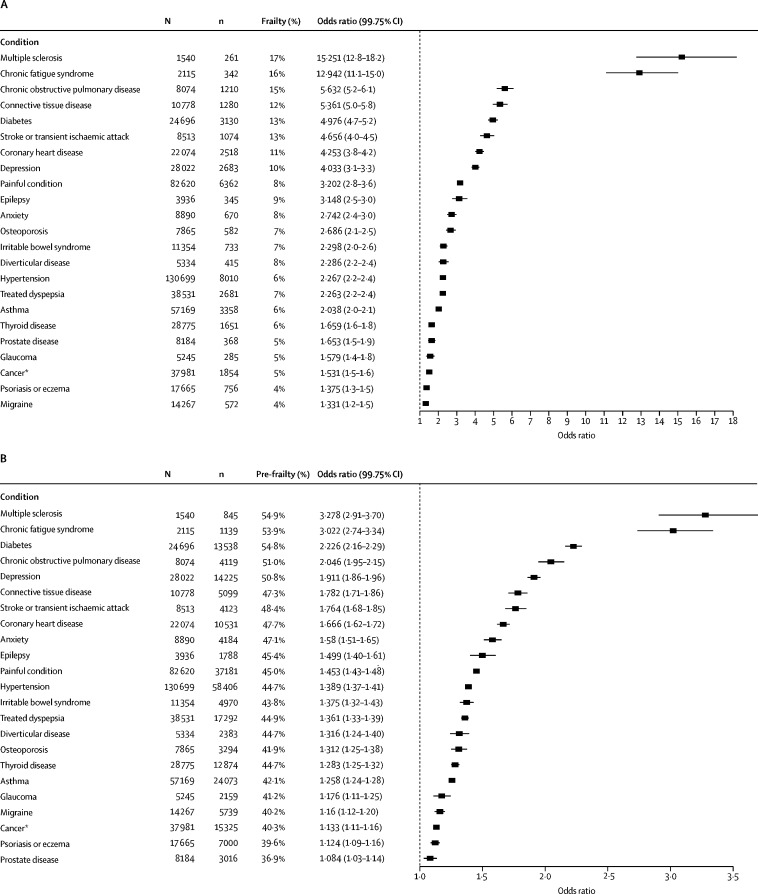
Frailty or pre-frailty for individual long-term conditions adjusted for age, sex, socioeconomic status, smoking status, and body-mass index A forest plot indicating the odds ratio for frailty (A) and pre-frailty (B) for long-term conditions with >1% prevalence (in the whole cohort) compared those without. *Excludes non-melanoma skin cancer.

Each individual long-term condition was associated with significantly greater odds of frailty and pre-frailty (adjusted for age, sex, socioeconomic status, body-mass index, smoking status, and alcohol use frequency; figure 2A and B). The associations for multiple sclerosis, chronic fatigue syndrome, connective tissue disease, diabetes, and chronic obstructive pulmonary disease with frailty had the largest effect sizes.

At the end of follow-up, 13 801 (3%) of 493 737 participants had died (1398 [8%] of 16 538 in the frail group, 6366 [3%] of 185 360 in the pre-frail group, and 6037 [2%] of 291 839 in the non-frail group). Mortality was higher in those with multimorbidity (7412 [5%] of 161 576 with at least two long-term conditions, 6330 [2%] of 330 547 with one or fewer long-term conditions). Each frailty indicator was independently associated with a higher risk of mortality in all age and sex groups except females aged 37–45 years, and overall risk was higher with increasing number of frailty indicators (appendix).

**Figure 3:**
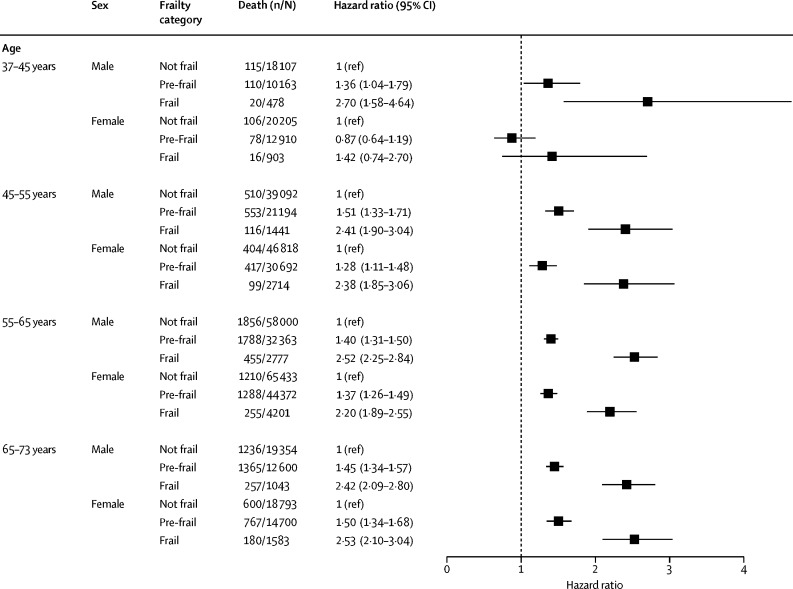
All-cause mortality for frailty status stratified by age and sex, and adjusted for socioeconomic status, body-mass index, smoking status, alcohol use frequency, and multimorbidity count

Analysis of the mortality associated with frailty and pre-frailty, adjusted for multimorbidity count, socio-economic status, body-mass index, smoking status, and alcohol use are shown in figure 3. Both frailty and pre-frailty were significantly associated with mortality for all strata except in women aged 37–45 years. In the subgroup analyses, the effect size was greater in current or previous smokers, in weekly or more frequent alcohol drinkers, and those with body-mass index of more than 25 (particularly in women aged 45–55 years) but it did not vary for different multimorbidity or socioeconomic status categories (appendix).

## Discussion

In this prospective cohort comprising nearly half a million middle-aged and older-aged people, our findings show, by use of an adaptation of the frailty phenotype, that frailty and pre-frailty are associated with mortality in men of all ages included (37–73 years) and in women aged 45–73 years. This is the first attempt to adapt the frailty phenotype criteria to the UK Biobank cohort, which includes a considerably younger age range than previous studies. Frailty was strongly associated with multi-morbidity; however, the associated mortality risk persisted after controlling for the number of long-term conditions. Our adapted definition identified 16 538 frail individuals (3%) of 493 737 study participants, and over 185 000 (38%) who met the criteria for pre-frailty. The prevalence of frailty was high in people with long-term conditions (eg, 17% in multiple sclerosis). Frailty and pre-frailty were associated with age, female sex, both obesity and underweight, smoking, socioeconomic deprivation, and infrequent alcohol intake. Frailty was associated with a more than two-times increased risk of mortality in men and women aged 45 years and older and men aged 37–45 years; however, the absolute rate of mortality was considerably higher in participants with multimorbidity. These findings are important given growing levels of multimorbidity affecting younger, socioeconomically deprived populations.^1^ The association of frailty with increased mortality—independently of recognised risk factors and number of long-term conditions—has important implications for risk stratification, clinical guidelines, health-care delivery, and design and planning of interventions to address increasing multimorbidity. Assessment of frailty in people with multimorbidity might facilitate identification of those at greatest risk, who would benefit most from intervention.

The existing frailty literature has not focused sufficiently on people aged younger than 65 years, with studies generally involving selected populations of younger people or including a wider age range in which the number of frail people aged younger than 65 years is unclear.^14^, ^16^, ^18^, ^19^ The largest study involving people younger than age 65 years included 36 751 participants aged at least 50 years from the longitudinal Survey of Health, Ageing and Retirement in Europe (1528 were frail, mean age 73 years).^23^ This study showed that the discrimination ability of the comorbidity index and frailty to predict mortality decreased with increasing age. Our findings suggest assessment of frailty might help to identify high-risk, multimorbid patients; however, the concept of frailty should be applied with caution to younger patients. Although associated with mortality, the adapted phenotypic definition of frailty used here might be clinically and biologically different from that encountered in older patients.^6^ Greater understanding of the implications of this phenotype across different ages, and in a range of long-term conditions, is required. Interventions to reverse frailty or improve outcomes have almost exclusively focused on the elderly or people in long-term care.^5^ Our findings indicate a need for a change in focus, to identify frailty and intervene much earlier. Clinicians and policy makers require an evidence base regarding what interventions might be effective in reducing frailty, or ameliorating its effects. Future research should explore the consequences of frailty across a wider age range and in those affected by multimorbidity. This will inform intervention development, which should be targeted at modifying or reversing the process. Interventions should be tailored to patients' clinical contexts, as no single intervention is likely to be applicable to all those meeting the criteria for frailty.^5^ By intervening earlier, individuals and health-care systems have potentially more to gain.

The high proportion of people identified as pre-frail suggests that screening of younger populations for pre-frailty is probably unhelpful, because such an approach is unlikely to have sufficient specificity. Instead, we suggest that an assessment of frailty should be incorporated into routine monitoring and assessment of people with multiple morbidity, which might help identification of those at greater risk to ensure more accurate targeting of the multidimensional, patient-centred reorganisation of care required to address complex multimorbidity, which has been highlighted as an important issue.^4^ Such an approach is likely to identify a higher proportion of frail people and use fewer resources through the use of existing structure and practices and could be incorporated into current chronic disease management pathways. An assessment of frailty should complement, rather than replace, a holistic assessment of a patient's needs and preferences.

Access to health care is more difficult for people in socioeconomically deprived areas who have higher levels of multimorbidity.^24^ The strong association between frailty, socioeconomic deprivation, and multimorbidity shown here has implications for the distribution of health-care resources, which should be targeted at those with greatest clinical need to avoid further increasing inequalities. The configuration of health-care delivery, which is currently highly fragmented and difficult to navigate, increases treatment burden experienced by patients and needs to be re-imagined in ways that will better support these high risk individuals and increase their capacity to self-manage to maximise their own wellbeing.^25^

Further research should explore the implications of frailty in younger people within specific clinical contexts. There is also a pressing need for public engagement on this subject and research to explore understanding and beliefs about frailty and frailty prevention. Such work is needed to inform design of interventions to identify, prevent, and manage frailty and pre-frailty across the age spectrum, particularly in those with multimorbidity.

To our knowledge, this is the largest longitudinal study to assess the effect of frailty on mortality, and the number of younger participants (those aged younger than 65 years) is far greater than in previous studies. We were able to adjust our analyses for a wide range of sociodemographic and lifestyle factors and multimorbidity. However, these data were only available at baseline and we were unable to model changes in multimorbidity or frailty over time, which could be considered a limitation. Other potentially important sociodemographic factors associated with frailty, such as diet, were not included in the model. The finding that participants who never or only occasionally drank alcohol were more likely to be frail was surprising, possibly explained by abstainer bias (people may drink no alcohol because they have poorer health and might have been advised not to).^26^

UK Biobank is a non-probability sample (ie, participants had to respond to an invitation to be included and were not completely random). Participants are mostly white British and comparatively less socioeconomically deprived than the UK average.^27^ As such, direct inferences cannot be made concerning the prevalence of frailty in the wider population and our findings are likely to be conservative.

Our adaptation of the frailty phenotype is based on a mixture of objective measurements and self-reported characteristics. Evaluations of self-reported frailty criteria have shown them to be equivalent,^28^ or in some cases superior,^29^ to measured alternatives; however, specific questions such as walking speed and weight loss might be affected by reporting bias. Several of our indicators also differ from the original frailty phenotype: importantly, we only had data on weight loss, not unintentional weight loss, which potentially underestimates the effects of this indicator as it could include people who lost weight deliberately.^6^ They might also have different implications across different age groups, and might not necessarily be markers of ageing when applied to younger people. Exhaustion, notably, was associated with younger age, and weight loss, low grip strength, and low physical activity were not associated with mortality in women aged 37–45 years. For this reason, in addition to combining them in the phenotype model, we assessed each frailty criterion separately in each age and sex category to support their application to this younger cohort. The use of self-reported data to identify long-term conditions is a limitation that potentially led to recall bias; however, participants were supported by a study nurse when providing these data and self-reported illness has been suggested as a valid measure of morbidity.^30^ The presence of shared characteristics might explain apparent associations between the frailty phenotype and some conditions (such as exhaustion or low-physical activity and chronic fatigue syndrome). In the absence of a gold-standard measure of multimorbidity,^31^ we considered a simple count, which was appropriate given the availability of data on a wide range of long-term conditions. However, our count was not weighted and we were unable to assess severity of the long-term conditions described. It also might have excluded potentially relevant conditions that might have been subclinical and not reported (eg, anaemia).

The frailty phenotype can be used to identify middle-aged and older-aged people at increased risk of mortality. The increasing focus on frailty as a clinical entity worthy of identification and intervention should be broadened to include the large and increasing number of younger people with multimorbidity, to allow targeted intervention to those with the greatest complexity. The specific associations and implications of frailty in middle age need to be explored and understood to inform tailored and individualised health promotion and health-care delivery, particularly in people with multimorbidity. Our study also suggests an urgent need to re-imagine and reconfigure health-care services to better meet the needs of our increasingly complex patient populations, because the status quo in untenable.

For the **hydraulic hand dynamometer** see http://biobank.ctsu.ox.ac.uk/crystal/refer.cgi?id=100232

For **STROBE guidelines** see https://www.strobe-statement.org

For **UK Biobank data** see www.ukbiobank.ac.uk
