# Supplementary material for: Frailty and pre-frailty in middle-aged and older adults and its association with multimorbidity and mortality: a prospective analysis of 493 737 UK Biobank participants
Source: Lancet Public Health. 2018 Jun 14;3(7):e323–32. doi: 10.1016/S2468-2667(18)30091-4 (PMC6028743; doi:10.1016/S2468-2667(18)30091-4)
Supplement: Supplementary appendix [file mmc1.pdf]

THE LANCET
Public Health

Supplementary appendix

This appendix formed part of the original submission and has been peer reviewed. We post it as supplied by the authors.

Supplement to: Hanlon P, Nicholl BI, Jani BD, Lee D, McQueenie R, Mair FS. Frailty and pre-frailty in middle-aged and older adults and its association with multimorbidity and mortality: a prospective analysis of 493 737 UK Biobank participants. *Lancet Public Health* 2018; published online June 13. http://dx.doi.org/10.1016/S2468-2667(18)30091-4.

Supplementary Appendix – Contents

Sociodemographic characteristics and frailty status

Descriptive cross-sectional analysis of baseline variables by frailty status Page 2

Multinomial model using age as a continuous variable Page 3

Validation of individual frailty indicators

Association between specific frailty indicators and mortality – univariate analyses Page 4

Association between increasing number of frailty indicators and mortality Page 5

Subgroup analyses – Cox Proportional Hazards models – association between frailty status and mortality – stratified by age and sex

Multimorbidity

Participants with 0 or 1 long-term conditions only Page 6

Participants with 2 or more long-term conditions Page 6

Socioeconomic status (assessed using Townsend score)

Most affluent 50% of participants Page 7

Most deprived 50% of participants Page 7

Body Mass Index

Participants with BMI <25 Page 8

Participants with BMI >25 Page 8

Smoking status

Never smokers only Page 9

Current or Previous smokers Page 9

Alcohol Frequency

Never or special occasions only Page 10

1-4 times weekly Page 10

Daily or almost daily drinkers Page 11

Relationship between frailty prevalence and number of LTCs

Analysis stratified by age and sex Page 12

Categorisation Long-Term conditions

Table of definitions of self-reported long-term conditions Page 13

Descriptive cross sectional analysis of baseline variables

Characteristic	Not frail (n=291,839)		Pre-frail (n=185,360)		Frail (n=16,538)		
	Count	%	Count	%	Count	%	p-value
Sex							
Male	138,270	47.4	80,136	43.2	6,587	39.8	
Female	153,569	52.6	105,224	56.8	9,951	60.2	<0.001
Age							
37-45 years	38,533	13.2	23,261	12.5	1,417	8.6	
45-55 years	86,824	29.8	52,856	28.5	4,370	26.4	
55-65 years	126,499	43.3	79,811	43.1	7,688	46.5	
65-73 years	39,983	13.7	29,432	15.9	3,063	18.5	
Median (IQR)	58 (50-63)		58 (50-63)		59 (52-64)		<0.001
Ethnicity							
White	280,563	96.4	170,982	92.6	14,448	87.9	
Other	10,400	3.6	13,634	7.4	1,995	12.1	<0.001
Missing	876		744		95		
Socioeconomic deprivation quintile							
1 (least deprived)	65,753	22.6	32,221	17.4	1,545	9.4	
2	63,229	21.7	33,673	18.2	1,915	11.6	
3	60,275	20.7	36,046	19.5	2,455	14.9	
4	56,311	19.3	38,740	20.9	3,642	22.1	
5 (most deprived)	45,971	15.8	44,396	24.0	6,952	42.1	<0.001
Missing	300		284		29		
Household income							
< £18,000	45,146	17.8	43,616	28.0	7,013	55.7	
£18,000 – 30,999	63,015	24.8	41,667	26.7	2,925	23.2	
£31,000 – 51,999	70,527	27.8	38,118	24.5	1,730	13.7	
£52,000 – 100,000	58,964	23.2	26,246	16.8	798	6.3	
> £100,000	16,496	6.5	6,237	4.0	130	1.0	<0.001
Missing	37,691		29,476		3,942		
University degree							
No	183,944	63.5	130,752	71.5	13,600	84.0	
Yes	105,589	34.5	52,176	28.5	2,589	16.0	<0.001
Missing	2,306		2,432		349		
Smoking status							
Current	25,888	8.9	22,513	12.2	3,312	20.2	
Previous	100,301	34.5	64,646	35.0	5,653	34.5	
Never	164,772	56.6	97,373	52.8	7,436	45.3	<0.001
Missing	878		828		137		
Alcohol frequency							
Daily	66,494	22.8	32,293	17.4	1,739	10.5	
1-4 times/week	152,120	52.2	84,291	45.5	5,181	31.4	
1-3 times/month	30,315	10.4	22,957	12.3	1,957	11.9	
Occasional/Never	42,766	14.7	45,699	24.7	7,610	46.2	<0.001
Missing	144		263		51		
BMI							
<18.5	1,371	0.5	1,034	0.6	150	0.9	
18.5-24.9	106,856	36.7	46,388	25.3	2,449	15.4	
25.0-29.9	129,710	44.6	77,153	42.0	4,984	31.4	
>30	53,218	18.3	58,919	32.1	8,279	52.2	<0.001
Missing	684		1,866		676		
Multimorbidity							
0 LTCs	117,531	40.4	50,187	27.2	1,397	8.5	
1 LTC	99,227	34.1	59,082	32.0	3,123	19.1	
2 LTCs	40,726	16.9	40,726	22.1	3987	24.3	
3LTCs	17,997	6.2	20,941	11.3	3443	21.0	

≥4 LTCs<br>Missing<br><br>Median (IQR)	7,299<br>641<br><br>1 (0-2)	2.5	13,604<br>820<br><br>1 (0-2)	7.4	4,435<br>153<br><br>2 (1-4)	27.1	<0.001<br><br><br><0.001

Multinomial model using age as a continuous variable

Pre-frail vs. Not Frail			Frail vs. Not Frail		
Predictor	Odds ratio	95% CI	Predictor	Odds ratio	95% CI
Sex<br>Male<br>Female	 (ref)<br>1·18	 (ref)<br>1·17 – 1·20	Sex<br>Male<br>Female	 (ref)<br>1·21	 (ref)<br>1·17 – 1·26
Age<br>OR per 1 year increase	0.999	0.998-0.999	Age<br>OR per 1 year increase	1.002	1.000-1.004
Socioeconomic deprivation quintile<br>1 (least deprived)<br>2<br>3<br>4<br>5 (most deprived)	 (ref)<br>1·05<br>1·15<br>1·27<br>1·62	 (ref)<br>1·03 – 1·07<br>1·13 – 1·17<br>1·25 – 1·30<br>1·59 – 1·65	Socioeconomic deprivation quintile<br>1 (least deprived)<br>2<br>3<br>4<br>5 (most deprived)	 (ref)<br>1·19<br>1·48<br>2·07<br>3·71	 (ref)<br>1·11 – 1·27<br>1·38 – 1·58<br>1·94 – 2·21<br>3·50 – 3·94
Smoking status<br>Never<br>Previous<br>Current	 (ref)<br>1·05<br>1·42	 (ref)<br>1·03 – 1·06<br>1·39 – 1·45	Smoking status<br>Never<br>Previous<br>Current	 (ref)<br>1·07<br>2·46	 (ref)<br>1·03 – 1·12<br>2·34 – 2·58
Alcohol frequency<br>Daily<br>1-4 times/week<br>1-3 times/month<br>Occasional/Never	 0·88<br>(ref)<br>1·23<br>1·58	 0·87 – 0·90<br>(ref)<br>1·20 – 1·25<br>1·56 – 1·61	Alcohol frequency<br>Daily<br>1-4 times/week<br>1-3 times/month<br>Occasional/Never	 0·75<br>(ref)<br>1·48<br>3·05	 0·71 – 0·80<br>(ref)<br>1·40 – 1·57<br>2·92 – 3·18
BMI<br><18.5<br>18.5-24.9<br>25.0-29.9<br>>30	 1·48<br>(ref)<br>1·35<br>2·18	 1·36 – 1·61<br>(ref)<br>1·33 – 1·37<br>2·14 – 2·22	BMI<br><18.5<br>18.5-24.9<br>25.0-29.9<br>>30	 2·92<br>(ref)<br>1·50<br>4·03	 2·42 – 3·55<br>(ref)<br>1·43 – 1·58<br>3·84 – 4·24
Multimorbidity<br>0 LTCs<br>1 LTC<br>2 LTCs<br>3 LTCs<br>≥4 LTCs	 (ref)<br>1·32<br>1·72<br>2·26<br>3·32	 (ref)<br>1·30 – 1·34<br>1·69 – 1·75<br>2·20 – 2·31<br>3·22 – 3·43	Multimorbidity<br>0 LTCs<br>1 LTC<br>2 LTCs<br>3 LTCs<br>≥4 LTCs	 (ref)<br>2·27<br>5·14<br>10·41<br>27·29	 (ref)<br>2·12 – 2·42<br>4·82 – 5·49<br>9·73 – 11·15<br>25·45 – 29·26
All covariates highlighted in bold are statistically significant (p<0·001)<br>*p = 0·0012, **p = 0·08<br>Results based on n=488,087 participants with complete data for all covariates (5650 (1·1%) missing data)					

Weight loss – univariate analysis – Cox PH model

Age	Females		Males	
	HR	95% CI	HR	95% CI
37-45	1.14	0.8-1.62	1.38	1.01-1.88
45-55	1.43	1.22-1.68	1.58	1.37-1.81
55-65	1.46	1.33-1.6	1.28	1.18-1.39
65-73	1.48	1.31-1.68	1.28	1.16-1.41

Low grip strength – univariate analysis – Cox PH model

Age	Females		Males	
	HR	95% CI	HR	95% CI
37-45	1.53	0.92-2.55	2.10	1.43-3.10
45-55	1.66	1.38-2.0	2.14	1.83-2.51
55-65	1.22	1.11-1.35	1.73	1.60-1.87
65-73	1.37	1.22-1.53	1.45	1.34-1.58

Low physical activity – univariate analysis – Cox PH model

Age	Females		Males	
	HR	95% CI	HR	95% CI
37-45	1.31	0.88-1.97	2.43	1.77-3.34
45-55	1.66	1.43-1.92	2.59	2.26-2.98
55-65	1.86	1.69-2.05	2.56	2.37-2.76
65-73	1.82	1.61-2.06	2.09	1.90-2.31

Self-reported exhaustion – univariate analysis – Cox PH model

Age	Females		Males	
	HR	95% CI	HR	95% CI
37-45	1.49	1.08-2.06	1.93	1.43-2.61
45-55	1.66	1.43-1.92	2.05	1.78-2.35
55-65	1.63	1.48-1.80	1.90	1.75-2.06
65-73	1.67	1.45-1.92	1.95	1.75-2.17

Self-reported slow walking speed – univariate analysis – Cox PH model

Age	Females		Males	
	HR	95% CI	HR	95% CI
37-45	3.18	2.13-4.74	3.57	2.46-5.18
45-55	3.12	2.63-3.67	4.00	3.46-4.63
55-65	2.61	2.38-2.87	3.35	3.12-3.59
65-73	2.43	2.16-2.73	2.60	2.39-2.83

Cox PH model. Mortality risk associated with increasing number of frailty indicators.

Number of frailty indicators	Hazard Ratios (95% confidence intervals)								
	All participants (age/sex adjusted) (n = 493,737)	Females				Males			
		37-45 years (n=34,218)	45-55 years (n=81,144)	55-65 years (n=116,759)	65-73 years (n=36,623)	37-45 years (n=28,993)	45-55 years (n=62,906)	55-65 years (n=97,239)	65-73 years (n=35,855)
**0 indicators**	(ref)	(ref)	(ref)	(ref)	(ref)	(ref)	(ref)	(ref)	(ref)
**1 indicator**	1.44 (1.39 - 1.50)	0.96 (0.69 – 1.34)	1.36 (1.17 – 1.59)	1.40 (1.29 – 1.53)	1.46 (1.30 – 1.65)	1.49 (1.12 – 1.99)	1.62 (1.42 – 1.86)	1.44 (1.34 – 1.55)	1.47 (1.35 – 1.60)
**2 indicators**	2.48 (2.36 – 2.60	1.91 (1.25 – 2.92)	2.33 (1.92 – 2.82)	2.13 (1.91 – 2.38)	2.15 (1.86 – 2.48)	2.78 (1.89 – 4.09)	3.58 (3.04 – 4.22)	2.77 (2.53 – 3.03)	2.44 (2.19 – 2.71)
**3 indicators**	3.71 (3.46 – 3.96)	3.24 (1.78 – 5.89)	3.90 (3.03 – 5.02)	3.06 (2.62 – 3.56)	3.26 (2.70 – 3.94)	6.10 (3.56 – 10.45)	5.15 (4.04 – 6.55)	4.55 (4.04 – 5.12)	3.07 (2.63 – 3.60)
**4 indicators**	5.37 (4.82 – 5.97)	4.10 (1.51 – 11.13)	5.64 (3.79 – 8.38)	3.77 (2.89 – 4.91)	4.03 (2.97 – 5.47)	9.62 (3.93 – 23.56)	8.47 (5.96 – 12.05)	6.08 (5.04 – 7.34)	6.15 (4.86 – 7.79)
**5 indicators**	6.13 (4.59 – 8.19)	-	4.03 (1.01 – 16.17)	5.79 (3.12 – 10.79)	3.65 (1.37 – 9.75)	-	15.45 (7.33 – 32.58)	6.69 (4.02 – 11.12)	5.58 (2.79 – 11.19)

Multimorbidity – subgroup analyses – '0-1 comorbidities' versus '2 or more'

MMcount 0 or 1 – Hazard ratios of all–cause mortality for frailty status stratified by age and sex, adjusted for socioeconomic status, BMI, smoking and alcohol frequency

Age Category	Sex	Frailty Category	Total	No. of deaths	Lower	95% CI HR	Upper
37–45 years	Male	Not frail	16034	89		(ref)	
		Pre–frail	8098	59	0.9	1.25	1.76
		Frail	252	5	1.09	2.74	6.88
	Female	Not frail	17393	75		(ref)	
		Pre–Frail	9690	37	0.56	0.84	1.26
		Frail	426	3	0.26	1.06	4.36
45–55 years	Male	Not frail	32412	362		(ref)	
		Pre–frail	14759	265	1.23	1.44	1.7
		Frail	523	21	1.57	2.49	3.93
	Female	Not frail	37865	282		(ref)	
		Pre–frail	20655	225	1.14	1.36	1.63
		Frail	928	20	1.63	2.59	4.11
55–65 years	Male	Not frail	41020	1114		(ref)	
		Pre–frail	17699	703	1.23	1.36	1.5
		Frail	636	68	2.14	2.77	3.58
	Female	Not frail	45644	728		(ref)	
		Pre–frail	23761	531	1.21	1.35	1.52
		Frail	1061	44	1.7	2.34	3.22
65–73 years	Male	Not frail	11439	620		(ref)	
		Pre–frail	5622	439	1.2	1.36	1.54
		Frail	194	41	2.28	3.14	4.33
	Female	Not frail	11376	305		(ref)	
		Pre–frail	6452	274	1.31	1.55	1.83
		Frail	278	20	1.45	2.32	3.73

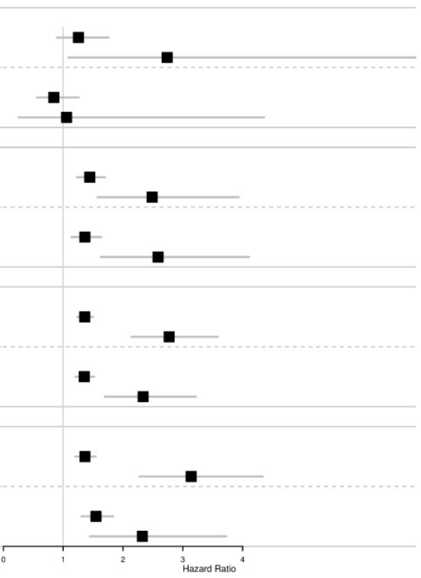

MMcount 2 or more – Hazard ratios of all–cause mortality for frailty status stratified by age and sex, adjusted for socioeconomic status, BMI, smoking and alcohol frequency

Age Category	Sex	Frailty Category	Total	No. of deaths	Lower	95% CI HR	Upper
37–45 years	Male	Not frail	2047	25		(ref)	
		Pre–frail	2013	51	1.13	1.86	3.06
		Frail	221	14	1.93	3.89	7.87
	Female	Not frail	2762	31		(ref)	
		Pre–Frail	3145	39	0.6	0.99	1.62
		Frail	464	13	0.87	1.86	3.96
45–55 years	Male	Not frail	6619	146		(ref)	
		Pre–frail	6354	284	1.46	1.8	2.21
		Frail	902	93	2.39	3.18	4.22
	Female	Not frail	8813	119		(ref)	
		Pre–frail	9857	191	1.05	1.33	1.69
		Frail	1755	78	2.05	2.81	3.85
55–65 years	Male	Not frail	16910	739		(ref)	
		Pre–frail	14566	1078	1.4	1.54	1.69
		Frail	2118	382	2.58	2.95	3.38
	Female	Not frail	19591	480		(ref)	
		Pre–frail	20408	747	1.3	1.47	1.65
		Frail	3105	210	2.09	2.49	2.97
65–73 years	Male	Not frail	7884	614		(ref)	
		Pre–frail	6947	918	1.42	1.58	1.75
		Frail	841	216	2.3	2.71	3.18
	Female	Not frail	7367	293		(ref)	
		Pre–frail	8181	492	1.33	1.54	1.78
		Frail	1294	159	2.31	2.84	3.48

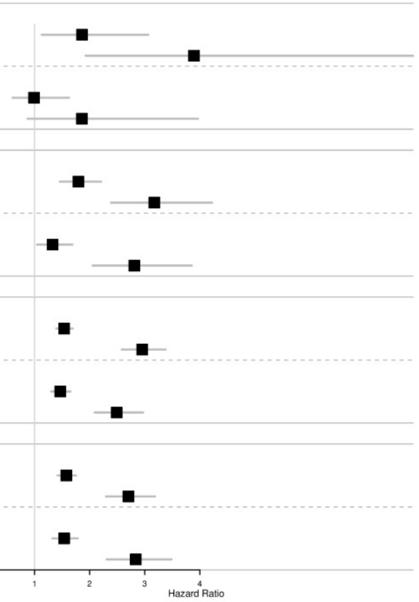

Socioeconomic status – most deprived 50% versus least deprived 50%

Most deprived 50% – Hazard ratios of all-cause mortality for frailty status stratified by age and sex, adjusted for multimorbidity count, BMI, alcohol frequency and smoking

Age Category	Sex	Frailty Category	Total	No. of deaths	Lower	95% CI HR	Upper
37–45 years	Male	Not frail	9802	67		(ref)	
		Pre–frail	6454	82	1.1	1.55	2.17
		Frail	392	19	1.86	3.32	5.93
	Female	Not frail	10642	65		(ref)	
		Pre–Frail	8059	55	0.57	0.84	1.24
		Frail	714	11	0.57	1.21	2.55
45–55 years	Male	Not frail	18881	283		(ref)	
		Pre–frail	12548	406	1.4	1.64	1.93
		Frail	1193	96	1.79	2.34	3.04
	Female	Not frail	22071	220		(ref)	
		Pre–frail	17447	261	1.01	1.22	1.47
		Frail	1959	75	1.62	2.19	2.97
55–65 years	Male	Not frail	24334	872		(ref)	
		Pre–frail	16961	1123	1.38	1.51	1.66
		Frail	2041	366	2.38	2.73	3.14
	Female	Not frail	28030	534		(ref)	
		Pre–frail	22670	719	1.25	1.41	1.59
		Frail	2819	190	1.88	2.27	2.73
65–73 years	Male	Not frail	7848	580		(ref)	
		Pre–frail	6450	782	1.27	1.42	1.59
		Frail	714	189	1.98	2.36	2.83
	Female	Not frail	8041	285		(ref)	
		Pre–frail	7512	421	1.26	1.48	1.73
		Frail	1029	114	1.79	2.28	2.9

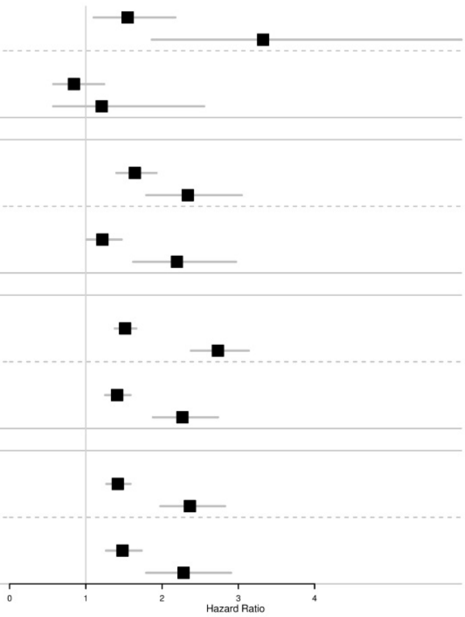

Most affluent 50% – Hazard ratios of all-cause mortality for frailty status stratified by age and sex, adjusted for multimorbidity count, BMI, alcohol frequency and smoking

Age Category	Sex	Frailty Category	Total	No. of deaths	Lower	95% CI HR	Upper
37–45 years	Male	Not frail	8271	48		(ref)	
		Pre–frail	3684	27	0.69	1.12	1.82
		Frail	84	1	0.16	1.19	9.07
	Female	Not frail	9534	41		(ref)	
		Pre–Frail	4811	23	0.55	0.93	1.58
		Frail	184	5	0.79	2.7	9.25
45–55 years	Male	Not frail	20166	226		(ref)	
		Pre–frail	8592	146	1.04	1.29	1.6
		Frail	245	19	2.38	3.94	6.52
	Female	Not frail	24702	184		(ref)	
		Pre–frail	13190	156	1.09	1.36	1.7
		Frail	749	23	1.75	2.77	4.38
55–65 years	Male	Not frail	33605	983		(ref)	
		Pre–frail	15369	662	1.18	1.31	1.45
		Frail	734	89	1.95	2.47	3.13
	Female	Not frail	37344	676		(ref)	
		Pre–frail	21648	569	1.19	1.34	1.5
		Frail	1376	65	1.58	2.07	2.72
65–73 years	Male	Not frail	11495	656		(ref)	
		Pre–frail	6141	580	1.34	1.5	1.68
		Frail	328	68	2.09	2.71	3.51
	Female	Not frail	10739	314		(ref)	
		Pre–frail	7182	346	1.29	1.52	1.78
		Frail	552	66	2.23	2.97	3.97

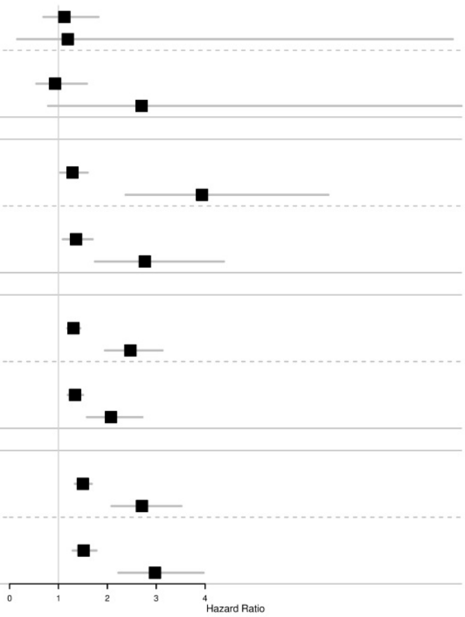

Body Mass Index – <25 versus >25

BMI <25 – Hazard ratios of all–cause mortality for frailty status stratified by age and sex, adjusted for multimorbidity count, socioeconomic status, alcohol frequency and smoking

Age Category	Sex	Frailty Category	Total	No. of deaths	Lower	95% CI HR	Upper
37–45 years	Male	Not frail	5689	42		(ref)	
		Pre–frail	2528	36	0.92	1.45	2.3
		Frail	97	10	2.33	5.13	11.26
	Female	Not frail	11126	59		(ref)	
		Pre–Frail	5179	29	0.55	0.87	1.37
		Frail	205	6	1.14	2.86	7.15
45–55 years	Male	Not frail	11040	132		(ref)	
		Pre–frail	4567	160	1.55	1.97	2.51
		Frail	253	35	2.13	3.26	4.99
	Female	Not frail	23457	197		(ref)	
		Pre–frail	10166	136	1.02	1.28	1.6
		Frail	476	29	2.25	3.45	5.28
55–65 years	Male	Not frail	15854	474		(ref)	
		Pre–frail	6215	399	1.43	1.64	1.89
		Frail	382	110	3.05	3.86	4.88
	Female	Not frail	29074	513		(ref)	
		Pre–frail	12587	376	1.26	1.44	1.65
		Frail	538	59	2.55	3.4	4.55
65–73 years	Male	Not frail	5371	308		(ref)	
		Pre–frail	2377	287	1.49	1.76	2.07
		Frail	127	71	3.33	4.45	5.96
	Female	Not frail	7904	253		(ref)	
		Pre–frail	3748	236	1.45	1.74	2.08
		Frail	225	45	2.12	3.01	4.28

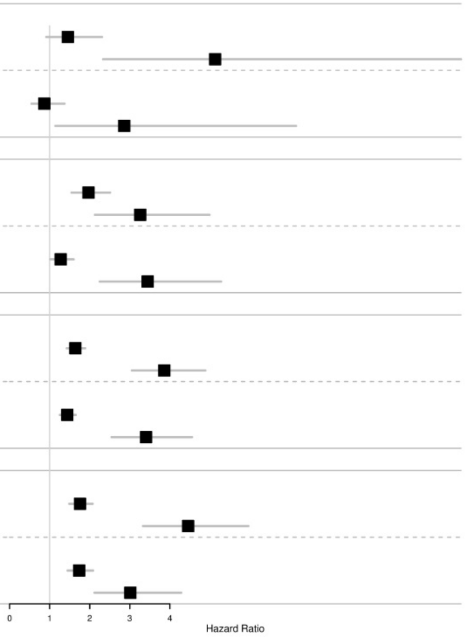

BMI >25 – Hazard ratios of all–cause mortality for frailty status stratified by age and sex, adjusted for multimorbidity count, socioeconomic status, alcohol frequency and smoking

Age Category	Sex	Frailty Category	Total	No. of deaths	Lower	95% CI HR	Upper
37–45 years	Male	Not frail	12396	71		(ref)	
		Pre–frail	7615	74	0.94	1.32	1.86
		Frail	380	9	0.9	1.89	3.96
	Female	Not frail	9063	47		(ref)	
		Pre–Frail	7713	48	0.62	0.94	1.42
		Frail	698	9	0.65	1.38	2.94
45–55 years	Male	Not frail	28010	377		(ref)	
		Pre–frail	16564	386	1.19	1.38	1.59
		Frail	1177	81	1.79	2.34	3.07
	Female	Not frail	23327	207		(ref)	
		Pre–frail	20477	279	1.09	1.31	1.57
		Frail	2227	70	1.59	2.14	2.89
55–65 years	Male	Not frail	42080	1376		(ref)	
		Pre–frail	26058	1377	1.25	1.35	1.46
		Frail	2381	342	2.05	2.34	2.67
	Female	Not frail	36289	694		(ref)	
		Pre–frail	31723	909	1.19	1.32	1.46
		Frail	3652	194	1.6	1.9	2.26
65–73 years	Male	Not frail	13959	927		(ref)	
		Pre–frail	10202	1074	1.26	1.38	1.51
		Frail	915	185	1.71	2.02	2.39
	Female	Not frail	10869	347		(ref)	
		Pre–frail	10929	528	1.21	1.39	1.6
		Frail	1357	133	1.9	2.36	2.91

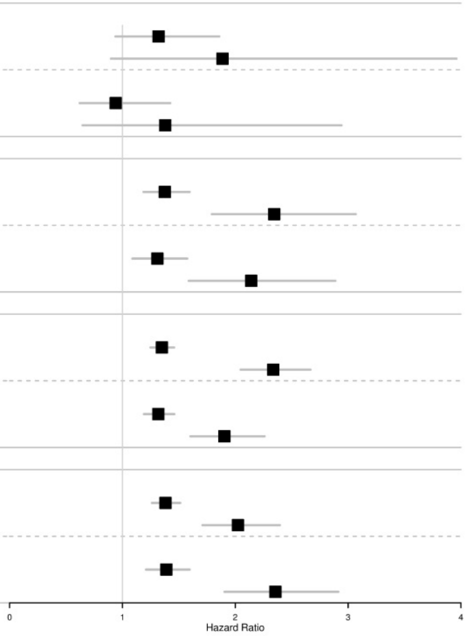

Smoking – Never versus Current or Previous

Never smokers – Hazard ratios of all−cause mortality for frailty status stratified by age and sex, adjusted for multimorbidity count, socioeconomic status, BMI, alcohol frequency

Age Category	Sex	Frailty Category	Total	No. of deaths	Lower	95% CI HR	Upper
37−45 years	Male	Not frail	11006	55		(ref)	
		Pre−frail	5663	50	0.91	1.36	2.04
		Frail	219	8	1.1	2.7	6.58
	Female	Not frail	12990	57		(ref)	
		Pre−Frail	7959	40	0.57	0.87	1.35
		Frail	520	6	0.35	1.01	2.93
45−55 years	Male	Not frail	22950	219		(ref)	
		Pre−frail	11357	198	1.2	1.47	1.8
		Frail	620	34	1.81	2.77	4.22
	Female	Not frail	29807	237		(ref)	
		Pre−frail	18337	205	0.94	1.15	1.4
		Frail	1461	31	1.04	1.58	2.4
55−65 years	Male	Not frail	27438	624		(ref)	
		Pre−frail	13739	487	1.19	1.35	1.53
		Frail	863	94	2.31	2.94	3.76
	Female	Not frail	38353	578		(ref)	
		Pre−frail	24801	558	1.22	1.38	1.56
		Frail	2202	97	1.72	2.19	2.78
65−73 years	Male	Not frail	8302	420		(ref)	
		Pre−frail	4812	360	1.17	1.35	1.56
		Frail	332	51	1.6	2.18	2.97
	Female	Not frail	11407	329		(ref)	
		Pre−frail	8464	343	1.14	1.34	1.56
		Frail	833	65	1.63	2.19	2.95

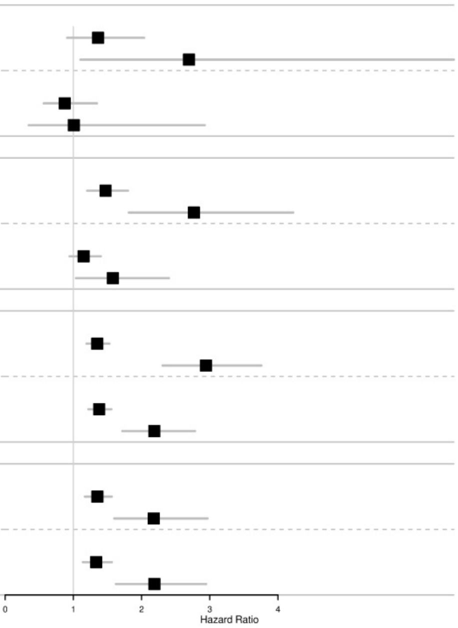

Current or previous smokers – Hazard ratios of all−cause mortality for frailty status stratified by age and sex, adjusted for multimorbidity count, socioeconomic status, BMI, alcohol frequency

Age Category	Sex	Frailty Category	Total	No. of deaths	Lower	95% CI HR	Upper
37−45 years	Male	Not frail	7061	59		(ref)	
		Pre−frail	4464	58	0.97	1.41	2.05
		Frail	259	12	1.65	3.23	6.31
	Female	Not frail	7168	49		(ref)	
		Pre−Frail	4923	38	0.55	0.86	1.34
		Frail	381	10	0.79	1.8	4.06
45−55 years	Male	Not frail	16062	288		(ref)	
		Pre−frail	9766	353	1.35	1.59	1.87
		Frail	816	82	1.91	2.53	3.35
	Female	Not frail	16906	166		(ref)	
		Pre−frail	12245	209	1.24	1.54	1.9
		Frail	1237	68	2.62	3.64	5.05
55−65 years	Male	Not frail	30369	1223		(ref)	
		Pre−frail	18468	1289	1.35	1.47	1.59
		Frail	1888	355	2.37	2.71	3.09
	Female	Not frail	26876	626		(ref)	
		Pre−frail	19361	720	1.27	1.42	1.59
		Frail	1971	153	2.02	2.45	2.97
65−73 years	Male	Not frail	10946	810		(ref)	
		Pre−frail	7696	1000	1.38	1.52	1.67
		Frail	694	200	2.24	2.64	3.12
	Female	Not frail	7312	268		(ref)	
		Pre−frail	6150	419	1.49	1.75	2.05
		Frail	725	114	2.45	3.11	3.96

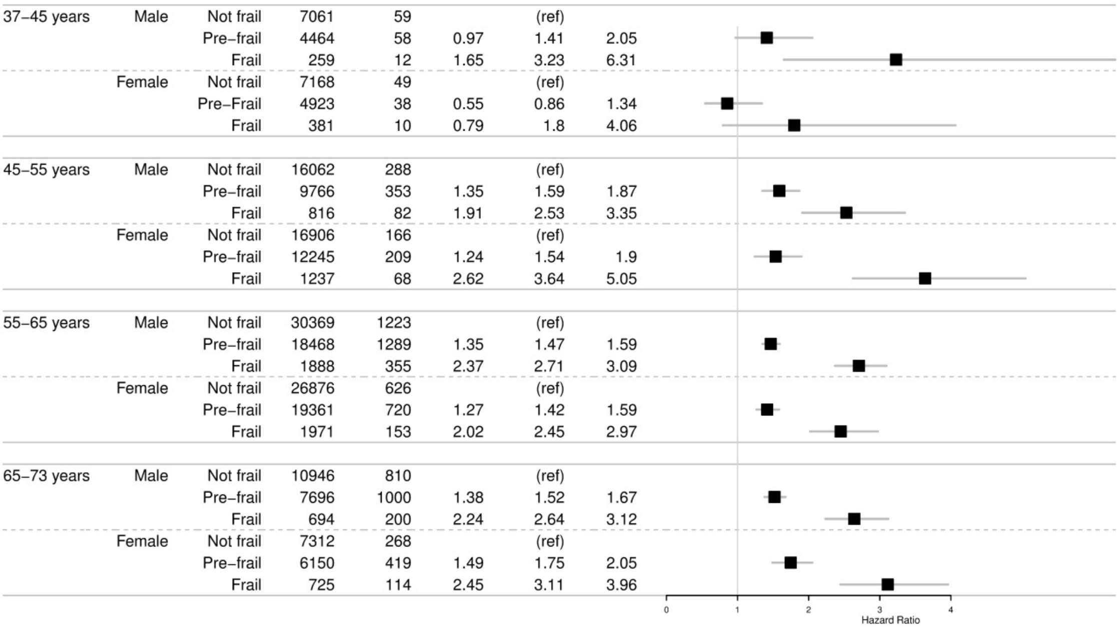

Alcohol frequency – never/occasional versus 1-4 per week versus daily

Never or special occasions only – Hazard ratios of all−cause mortality for frailty status stratified by age and sex, adjusted for multimorbidity count, socioeconomic status, BMI and smoking

Age Category	Sex	Frailty Category	Total	No. of deaths	Lower	95% CI HR	Upper
37−45 years	Male	Not frail	2084	13		(ref)	
		Pre−frail	2148	23	0.6	1.24	2.57
		Frail	213	11	1.5	3.88	10.03
	Female	Not frail	3509	26		(ref)	
		Pre−Frail	3667	31	0.46	0.8	1.4
		Frail	495	5	0.07	0.31	1.35
45−55 years	Male	Not frail	3817	63		(ref)	
		Pre−frail	3793	130	1.11	1.52	2.08
		Frail	576	45	1.38	2.14	3.3
	Female	Not frail	7657	81		(ref)	
		Pre−frail	8540	150	1.03	1.36	1.81
		Frail	1385	48	1.19	1.8	2.72
55−65 years	Male	Not frail	5369	211		(ref)	
		Pre−frail	5134	375	1.27	1.52	1.82
		Frail	945	156	1.93	2.45	3.12
	Female	Not frail	12341	284		(ref)	
		Pre−frail	13275	485	1.2	1.4	1.64
		Frail	2161	148	1.69	2.12	2.65
65−73 years	Male	Not frail	2260	170		(ref)	
		Pre−frail	2220	309	1.32	1.6	1.94
		Frail	345	106	2.01	2.63	3.45
	Female	Not frail	4694	187		(ref)	
		Pre−frail	5109	310	1.19	1.44	1.74
		Frail	863	108	1.76	2.29	3

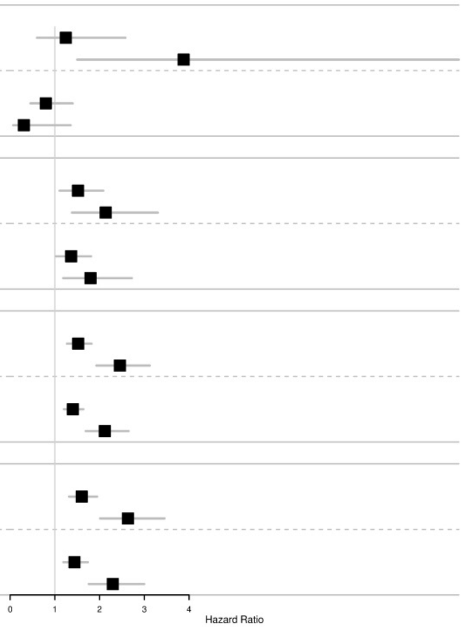

1−4 times per week – Hazard ratios of all−cause mortality for frailty status stratified by age and sex, adjusted for multimorbidity count, socioeconomic status, BMI and smoking

Age Category	Sex	Frailty Category	Total	No. of deaths	Lower	95% CI HR	Upper
37−45 years	Male	Not frail	10633	64		(ref)	
		Pre−frail	5152	47	0.85	1.26	1.86
		Frail	149	5	1.01	2.6	6.69
	Female	Not frail	10986	51		(ref)	
		Pre−Frail	5692	25	0.48	0.78	1.29
		Frail	220	8	1.54	3.66	8.75
45−55 years	Male	Not frail	22483	263		(ref)	
		Pre−frail	11007	236	1.21	1.46	1.76
		Frail	498	40	1.85	2.72	3.99
	Female	Not frail	25221	200		(ref)	
		Pre−frail	13660	166	1.04	1.29	1.6
		Frail	773	25	1.58	2.48	3.91
55−65 years	Male	Not frail	30241	928		(ref)	
		Pre−frail	16090	767	1.15	1.27	1.41
		Frail	1098	171	2.02	2.43	2.92
	Female	Not frail	31997	540		(ref)	
		Pre−frail	18664	463	1.16	1.32	1.5
		Frail	1204	56	1.45	1.95	2.63
65−73 years	Male	Not frail	9571	593		(ref)	
		Pre−frail	5967	572	1.17	1.31	1.48
		Frail	413	68	1.23	1.61	2.11
	Female	Not frail	8111	238		(ref)	
		Pre−frail	5524	259	1.18	1.42	1.71
		Frail	409	44	1.77	2.51	3.56

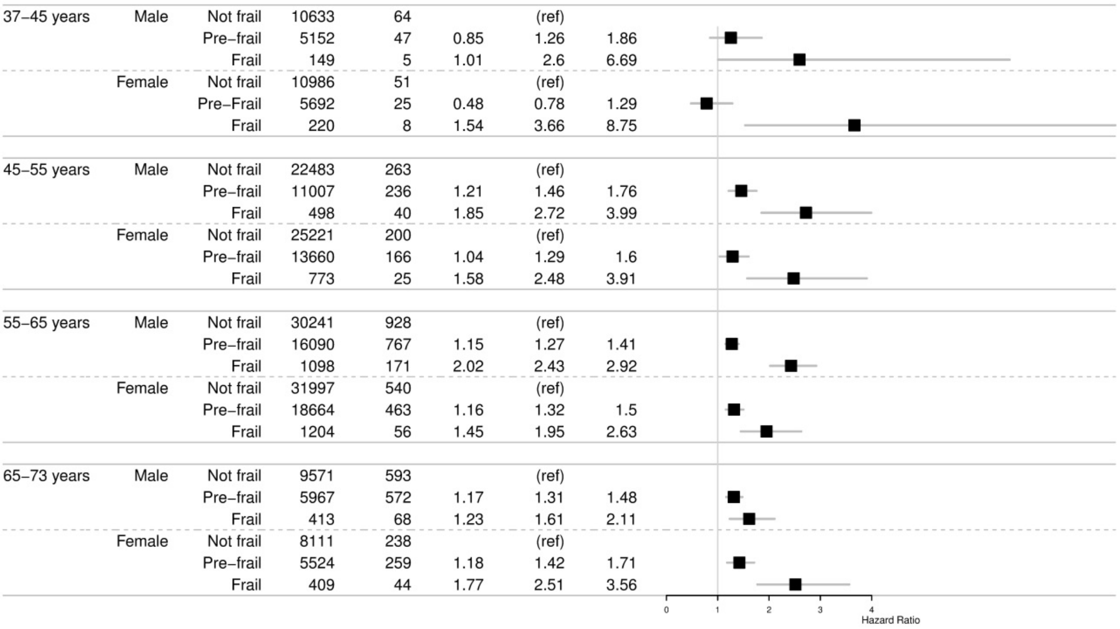

Daily or almost daily – Hazard ratios of all−cause mortality for frailty status stratified by age and sex, adjusted for multimorbidity count, socioeconomic status, BMI and smoking

Age Category	Sex	Frailty Category	Total	No. of deaths	95% CI Lower	HR	Upper
37−45 years	Male	Not frail	3285	21		(ref)	
		Pre−frail	1431	25	1.16	2.12	3.87
		Frail	58	3	0.56	2.15	8.22
	Female	Not frail	2569	13		(ref)	
		Pre−Frail	1353	5	0.25	0.71	2.05
		Frail	51	2	1.12	6.12	33.47
45−55 years	Male	Not frail	9352	138		(ref)	
		Pre−frail	4003	129	1.25	1.61	2.07
		Frail	181	19	1.35	2.35	4.11
	Female	Not frail	7958	73		(ref)	
		Pre−frail	3834	45	0.71	1.04	1.54
		Frail	164	12	2.38	4.74	9.45
55−65 years	Male	Not frail	18206	572		(ref)	
		Pre−frail	8183	480	1.36	1.55	1.76
		Frail	443	99	2.8	3.54	4.48
	Female	Not frail	13550	244		(ref)	
		Pre−frail	6489	189	1.13	1.38	1.69
		Frail	298	31	2.35	3.53	5.32
65−73 years	Male	Not frail	6162	392		(ref)	
		Pre−frail	3367	368	1.29	1.49	1.73
		Frail	184	55	2.31	3.12	4.21
	Female	Not frail	3852	107		(ref)	
		Pre−frail	2267	125	1.38	1.8	2.36
		Frail	123	16	1.78	3.15	5.57

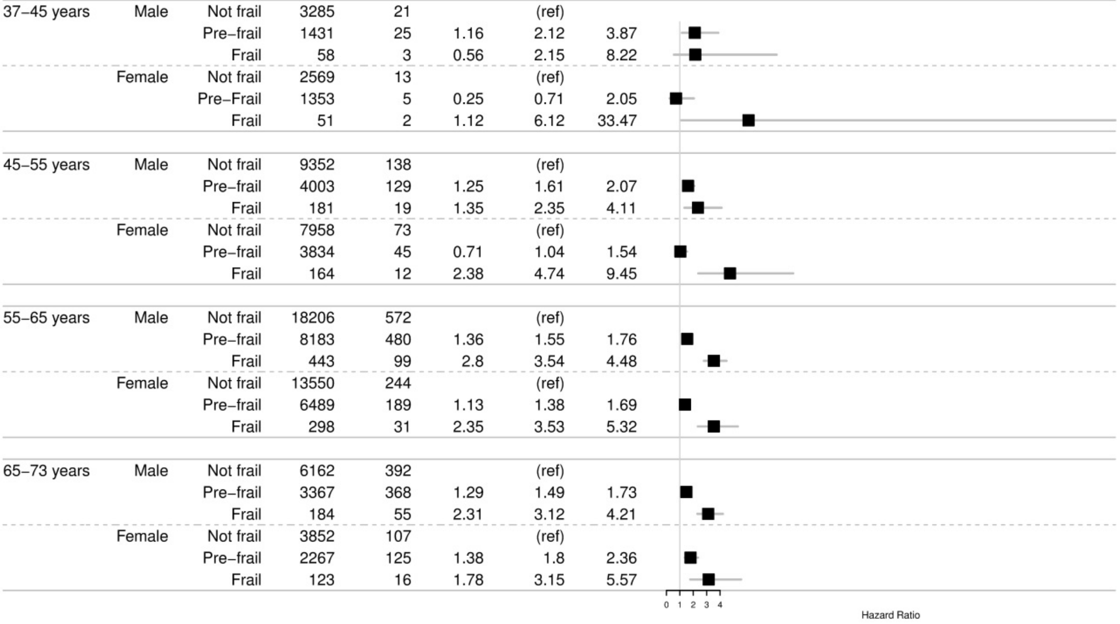

Relationship between frailty prevalence and number of LTCs: Analysis stratified by age and sex

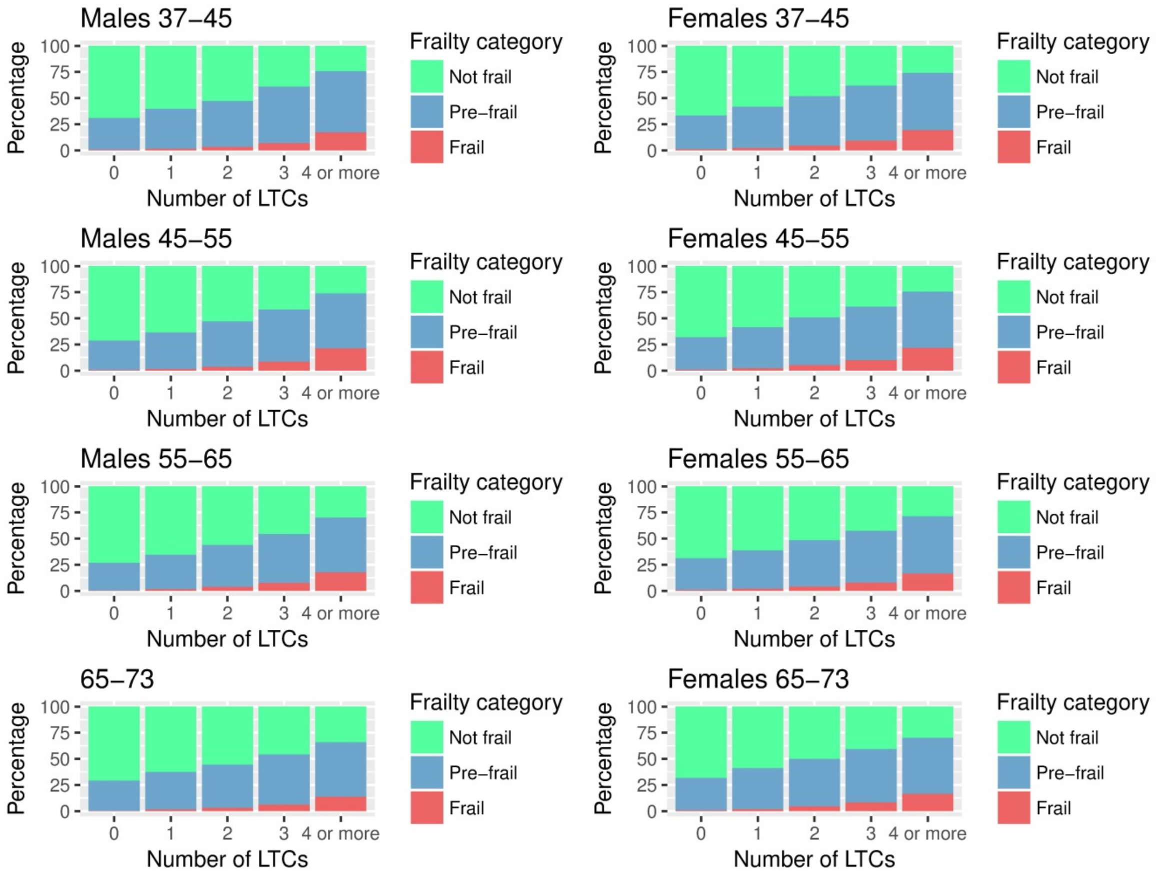

Categorisation of Long-Term Conditions

Morbidity grouping*	Conditions included
**Hypertension**	Hypertension<br>Essential hypertension
**Coronary heart disease**	Heart attack/MI<br>Angina
**Diabetes**	Diabetic nephropathy<br>Diabetic neuropathy/ulcers<br>Diabetes<br>Type 1 diabetes<br>Type 2 diabetes<br>Diabetic eye disease
**Stroke/TIA**	Stroke<br>TIA<br>Subarachnoid haemorrhage<br>Brain haemorrhage<br>Ischaemic stroke
**Atrial fibrillation**	Atrial fibrillation
**Heart failure**	Cardiomyopathy<br>Hypertrophic cardiomyopathy<br>Heart failure/pulmonary oedema
**Peripheral vascular disease**	Peripheral vascular disease<br>Leg claudication/intermittent claudication
**COPD**	COPD/Chronic obstructive pulmonary disease<br>Emphysema/Chronic bronchitis<br>Emphysema
**Asthma**	Asthma
**Bronchiectasis**	Bronchiectasis
**Cancer**	"yes"/"no" to "have you ever had cancer?"
**Dyspepsia**	Gastro-oesophageal reflux (GORD)<br>Oesophagitis/Barrett's oesophagus<br>Gastric stomach ulcers<br>Gastric erosions/gastritis<br>Duodenal ulcer<br>Dyspepsia/indigestion<br>Hiatus hernia<br>Helicobacter pylori
**Diverticular disease**	Diverticular disease/diverticulitis
**Irritable bowel syndrome**	Irritable bowel syndrome
**Chronic liver disease**	Oesophageal varices<br>Non infective hepatitis<br>Liver failure/cirrhosis<br>Primary biliary cirrhosis
**Inflammatory bowel disease**	Inflammatory bowel disease<br>Crohn's disease<br>Ulcerative colitis
**Constipation**	Constipation
**Viral hepatitis**	Hepatitis B<br>Hepatitis C<br>Hepatitis D

**Depression**	Depression<br>Postnatal depression
**Anxiety**	Anxiety/panic attacks<br>Nervous breakdown<br>Post-traumatic stress disorder<br>Obsessive compulsive disorder<br>Stress<br>Insomnia<br>Psychological/psychiatric problem
**Schizophrenia/Bipolar affective disorder**<br>**Bipolar**	Scizophrenia<br>Mania<br>Bipolar disorder<br>Manic depression
**Connective tissue diseases**	Myositis/myopathy<br>Systemic lupus erythematosus/SLE<br>Connective tissue disorder<br>Sjogren's syndrome.sicca syndrome<br>Dermatopolymyositis<br>Scloeroderma/systemic sclerosis<br>Rheumatoid arthritis<br>Psoriatic arthropathy<br>Dermatomyositis<br>Polymyositis<br>Polymyalgia rheumatica
**Painful conditions**	Back pain<br>Joint pain<br>Headaches (not migraine)<br>Sciatica<br>Plantar fasciitis<br>Carpal tunnel syndrome<br>Fibromyalgia<br>Arthritis<br>Shingles<br>Disc problem<br>Prolapsed disc/slipped disc<br>Spine arthritis/spondylitis<br>Ankylosing spondylitis<br>Back problem<br>Osteoarthritis<br>Gout<br>Cervical spondylosis<br>Trigeminal neuralgia<br>Disc degeneration<br>Trapped nerve/compressed nerve
**Osteoporosis**	Osteoporosis
**Thyroid disorders**	Thyroid problem (not cancer<br>Hyperthroidism/thyrotoxicosis<br>Hypothyroidism/myxoedema<br>Grave's disease<br>Thyroid goitre<br>Thyroitits

**Alcohol problems**	Alcohol dependency<br>Alcoholic liver disease/alcoholic cirrhosis
**Chronic kidney disease**	Polycystic kidney<br>Diabetic nephropathy<br>Renal/kidney failure<br>Renal failure requiring dialysis<br>Renal failure not requiring dialysis<br>Kidney nephropathy<br>Immunoglobulin A (IgA) nephropathy
**Prostate disorders**	Prostate problem (not cancer)<br>Enlarged prostate<br>Benign prostatic hypertrophy
**Glaucoma**	Glaucoma
**Epilepsy**	Epilepsy
**Dementia**	Dementia/Alzheimer/cognitive impairment
**Psoriasis or eczema**	Eczema/dermatitis<br>Psoriasis
**Migraine**	Migraine
**Chronic sinusitis**	Chronic sinusitis
**Anorexia or bulimia**	Anorexia, bulimia/other eating disorder
**Parkinson's disease**	Parkinson's disease
**Multiple sclerosis**	Multiple sclerosis
**Chronic fatigue syndrome**	Chronic fatigue syndrome
**Endometriosis**	Endometriosis
**Meniere disease**	Meniere disease
**Pernicious anaemia**	Pernicious anaemia
**Polycystic ovaries**	Polycystic ovaries
*Self-report lifetime diagnosis by doctor recorded by nurse-led interview (UK Biobank data field 20002), except cancer diagnosis which was reported by touch-screen questionnaire. The list of disease groupings was based on Barnett et al (2012)	
